# Supplementary material for: Investigation of Trehalose Supplementation Impacting Campylobacter jejuni and Clostridium perfringens from Broiler Farming
Source: Vet Sci. 2023 Jul 15;10(7):466. doi: 10.3390/vetsci10070466 (PMC10385778; doi:10.3390/vetsci10070466)
Supplement: Supplementary file 1 [file vetsci-10-00466-s001.zip › vetsci-2361830-supplementary.pdf]

Supplementary Materials

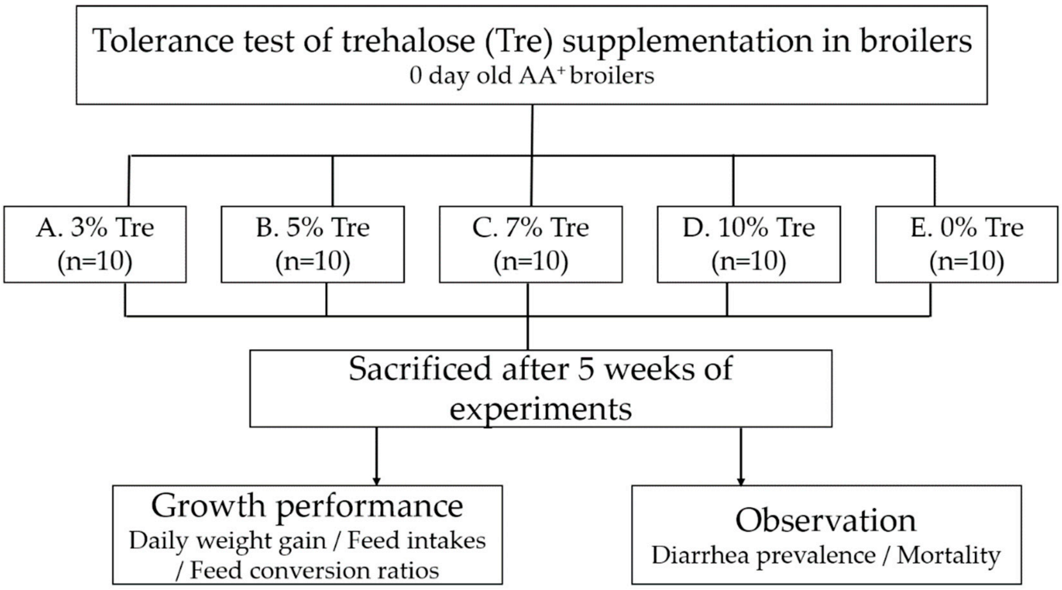

Supplementary Figure S1. Scheme of experiment 1: The tolerance test of trehalose on broilers

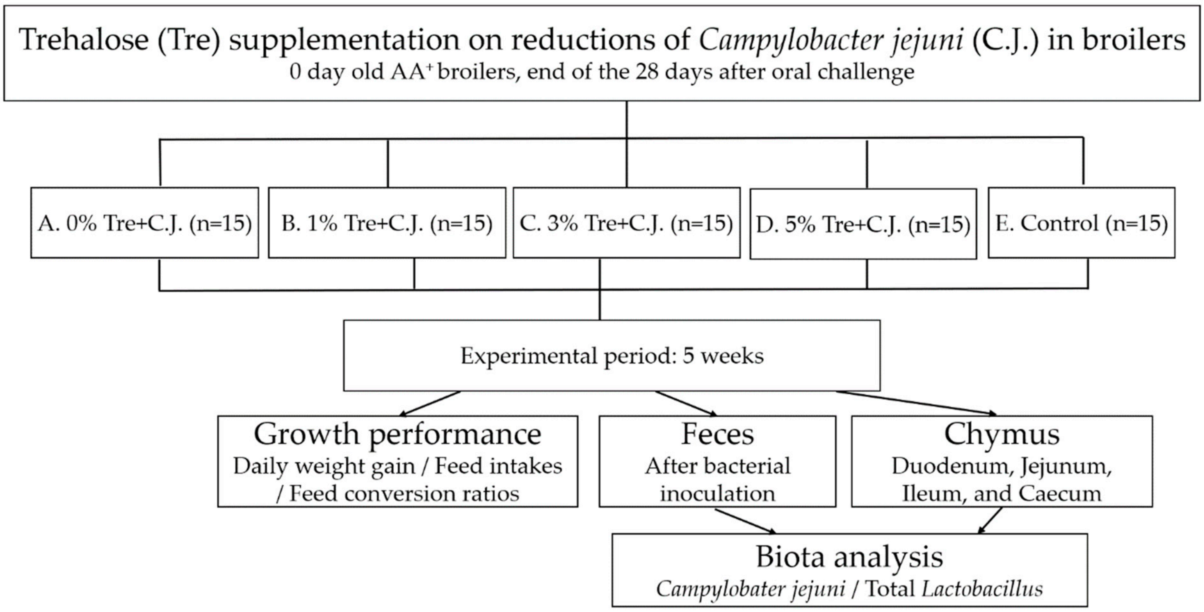

Supplementary Figure S2. Scheme of experiment 2-1 : The antibacterial tests of trehalose on *C. jejuni*

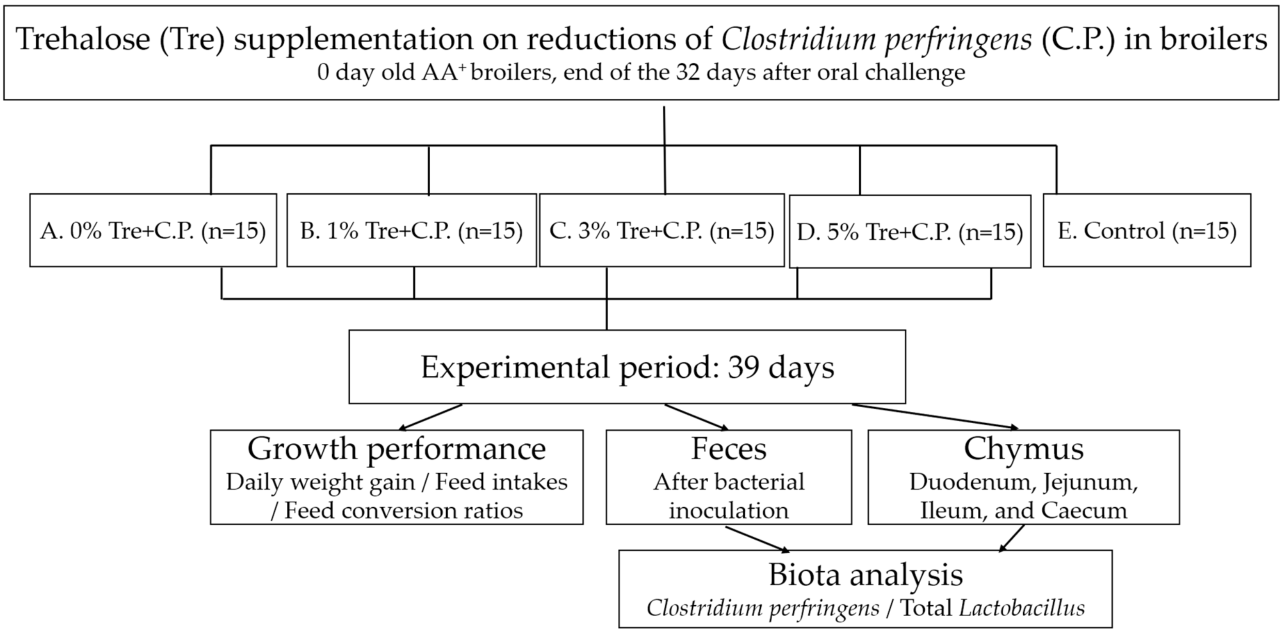

Supplementary Figure S3. Scheme of experiment 2-2 : The antibacterial tests of trehalose on *C. perfringens*

Supplementary Table S1. The components of experimental diets in the starter period of broilers.

	Levels of trehalose (%)				
**Ingredient**	**0**	**3**	**5**	**7**	**10**
			g/Kg		
Yellow corn	384.7	384.7	384.7	384.7	384.7
Soybean meal	398.1	398.1	398.1	398.1	398.1
SFish meal	50.0	50.0	50.0	50.0	50.0
Soybean oil	82.0	82.0	82.0	82.0	82.0
DL-methionine	2.0	2.0	2.0	2.0	2.0
$Ca(H_2PO_4)$-H_2O	13.1	13.1	13.1	13.1	13.1
$CaCO_3$	12.0	12.0	12.0	12.0	12.0
NaCl	3.0	3.0	3.0	3.0	3.0
Vitamin-premix	3.0	3.0	3.0	3.0	3.0
Mineral-premix	2.0	2.0	2.0	2.0	2.0
Choline	0.1	0.1	0.1	0.1	0.1
SiO_2	100.0	70.0	50.0	30.0	0.0
Trehalose	0.0	30.0	50.0	70.0	100.0
Total	1000.0	1000.0	1000.0	1000.0	1000.0
			Calculate value		
Metabolizable energy (Kcal/Kg)	3031	3031	3031	3031	3031
Crude protein (g/Kg)	235.0	235.0	235.0	235.0	235.0
Calcium (g/Kg)	10.9	10.9	10.9	10.9	10.9
Available phosphorus (g/Kg)	5.0	5.0	5.0	5.0	5.0

Supplementary Table S2. The components of experimental diets in the grower period of broilers.

Ingredient	Levels of trehalose (%)				
	**0**	**3**	**5**	**7**	**10**
			g/Kg		
Yellow corn	365.7	365.7	365.7	365.7	365.7
Soybean meal	440.6	440.6	440.6	440.6	440.6
Fish meal	0.0	0.0	0.0	0.0	0.0
Soybean oil	107.0	107.0	107.0	107.0	107.0
DL-methionine	2.3	2.3	2.3	2.3	2.3
$Ca(H_2PO_4)$-H_2O	16.0	16.0	16.0	16.0	16.0
$CaCO_3$	10.3	10.3	10.3	10.3	10.3
NaCl	3.0	3.0	3.0	3.0	3.0
Vitamin-premix	3.0	3.0	3.0	3.0	3.0
Mineral-premix	2.0	2.0	2.0	2.0	2.0
Choline	0.1	0.1	0.1	0.1	0.1
SiO_2	100.0	70.0	50.0	30.0	0.0
Trehalose	0.0	30.0	50.0	70.0	100.0
Total	1000.0	1000.0	1000.0	1000.0	1000.0
			Calculate value		
Metabolizable energy (Kcal/Kg)	3154	3154	3154	3154	3154
Crude protein (g/Kg)	220.0	220.0	220.0	220.0	220.0
Calcium (g/Kg)	11.8	11.8	11.8	11.8	11.8
Available phosphorus (g/Kg)	4.6	4.6	4.6	4.6	4.6

Supplementary Table S3. The components of experimental diets in the finisher period of broilers.

Ingredient	Levels of trehalose (%)				
	0	3	5	7	10
			g/Kg		
Yellow corn	393.2	393.2	393.2	393.2	393.2
Soybean meal	412.4	412.4	412.4	412.4	412.4
Fish meal	0.0	0.0	0.0	0.0	0.0
Soybean oil	109.0	109.0	109.0	109.0	109.0
DL-methionine	2.3	2.3	2.3	2.3	2.3
$Ca(H_2PO_4)$-H_2O	15.0	15.0	15.0	15.0	15.0
$CaCO_3$	10.0	10.0	10.0	10.0	10.0
NaCl	3.0	3.0	3.0	3.0	3.0
Vitamin-premix	3.0	3.0	3.0	3.0	3.0
Mineral-premix	2.0	2.0	2.0	2.0	2.0
Choline	0.1	0.1	0.1	0.1	0.1
SiO_2	100.0	70.0	50.0	30.0	0.0
Trehalose	0.0	30.0	50.0	70.0	100.0
Total	1000.0	1000.0	1000.0	1000.0	1000.0
			Calculate value		
Metabolizable energy (Kcal/Kg)	3201	3201	3201	3201	3201
Crude protein (g/Kg)	210.0	210.0	210.0	210.0	210.0
Calcium (%)	11.5	11.5	11.5	11.5	11.5
Available phosphorus (%)	4.3	4.3	4.3	4.3	4.3

Supplementary Table S4. The components of experimental diets in the starter period of broilers.

Ingredient	Levels of trehalose (%)			
	**0**	**1**	**3**	**5**
		g/Kg		
Yellow corn	384.7	384.7	384.7	384.7
Soybean meal	398.1	398.1	398.1	398.1
Fish meal	50.0	50.0	50.0	50.0
Soybean oil	82.0	82.0	82.0	82.0
DL-methionine	2.0	2.0	2.0	2.0
$Ca(H_2PO_4)$-H_2O	13.1	13.1	13.1	13.1
$CaCO_3$	12.0	12.0	12.0	12.0
NaCl	3.0	3.0	3.0	3.0
Vitamin-premix	3.0	3.0	3.0	3.0
Mineral-premix	2.0	2.0	2.0	2.0
Choline	0.1	0.1	0.1	0.1
SiO_2	50.0	40.0	20.0	0.0
Trehalose	0.0	10.0	30.0	50.0
Total	1000.0	1000.0	1000.0	1000.0
		Calculate value		
Metabolizable energy (Kcal/Kg)	3031	3031	3031	3031
Crude protein (g/Kg)	235.0	235.0	235.0	235.0
Calcium (g/Kg)	10.9	10.9	10.9	10.9
Available phosphorus (g/Kg)	5.0	5.0	5.0	5.0

Supplementary Table S5. The components of experimental diets in the grower period of broilers.

Ingredient	Levels of trehalose (%)			
	0	1	3	5
		g/Kg		
Yellow corn	365.7	365.7	365.7	365.7
Soybean meal	440.6	440.6	440.6	440.6
Fish meal	0.0	0.0	0.0	0.0
Soybean oil	107.0	107.0	107.0	107.0
DL-methionine	2.3	2.3	2.3	2.3
$Ca(H_2PO_4)$-H_2O	16.0	16.0	16.0	16.0
$CaCO_3$	10.3	10.3	10.3	10.3
NaCl	3.0	3.0	3.0	3.0
Vitamin-premix	3.0	3.0	3.0	3.0
Mineral-premix	2.0	2.0	2.0	2.0
Choline	0.1	0.1	0.1	0.1
SiO_2	50.0	40.0	20.0	0.0
Trehalose	0.0	10.0	30.0	50.0
Total	1000.0	1000.0	1000.0	1000.0
		Calculate value		
Metabolizable energy (Kcal/Kg)	3154	3154	3154	3154
Crude protein (g/Kg)	220.0	220.0	220.0	220.0
Calcium (g/Kg)	11.8	11.8	11.8	11.8
Available phosphorus (g/Kg)	4.6	4.6	4.6	4.6

Supplementary Table S6. The components of experimental diets in the finisher period of broilers.

Ingredient	Levels of trehalose (%)			
	**0**	**1**	**3**	**5**
	g/Kg			
Yellow corn	393.2	393.2	393.2	393.2
Soybean meal	412.4	412.4	412.4	412.4
Fish meal	0.0	0.0	0.0	0.0
Soybean oil	109.0	109.0	109.0	109.0
DL-methionine	2.3	2.3	2.3	2.3
$Ca(H_2PO_4)$-H_2O	15.0	15.0	15.0	15.0
$CaCO_3$	10.0	10.0	10.0	10.0
NaCl	3.0	3.0	3.0	3.0
Vitamin-premix	3.0	3.0	3.0	3.0
Mineral-premix	2.0	2.0	2.0	2.0
Choline	0.1	0.1	0.1	0.1
SiO_2	50.0	40.0	20.0	0.0
Trehalose	0.0	10.0	30.0	50.0
Total	1000.0	1000.0	1000.0	1000.0
	Calculate value			
Metabolizable energy (Kcal/Kg)	3201	3201	3201	3201
Crude protein (g/Kg)	210.0	210.0	210.0	210.0
Calcium (%)	11.5	11.5	11.5	11.5
Available phosphorus (%)	4.3	4.3	4.3	4.3

Supplementary Table S7. Effects of trehalose (Tre) on body weight (g) of broilers.

		Treatment				
**Age(day)**	**Aviagen[1]**	**Control**	**3% Tre**	**5% Tre**	**7% Tre**	**10% Tre**
0	42.00	44.81 ± 0.83	44.52 ± 0.70	44.53 ± 0.69	44.55 ± 0.73	44.84 ± 0.82
7	179.60	197.27 ± 1.96	188.27 ± 5.22	197.13 ± 3.16	182.46 ± 5.96	183.67 ± 5.13
14	450.00	543.07 ± 13.35	527.44 ± 14.21	548.70 ± 12.88	526.61 ± 16.06	535.64 ± 15.58
21	868.00	1066.43 ± 30.07	1035.15 ± 29.99	1067.04 ± 26.34	1052.59 ± 31.73	1056.83 ± 27.47
28	1406.00	1736.05 ± 54.28	1739.35 ± 49.14	1773.56 ± 50.48	1727.89 ± 52.90	1760.31 ± 47.04
35	2013.00	2462.38 ± 72.28	2502.37 ± 68.76	2608.17 ± 71.05	2493.47 ± 69.97	2497.07 ± 59.36

The data are given as mean±SEM (n = 14~15). Mean values with different letters within each feeding week are significantly different ($p < 0.05$). No significant differences ($p > 0.05$) were observed in body weight among the groups with 3%, 5%, 7% and 10% trehalose supplementation when compared to the control group throughout the test period. [1] Data are offered by the broiler feeding manual of Aviagen, a commercial broiler breeding company.

Supplementary Table S8. Effects of trehalose (Tre) on daily weight gain and feed intake, feed conversion ratio and mortality of broilers based on each feeding period.

Feeding period (day)	Parameter	Aviagen[1]	Control	3% Tre	Treatment 5% Tre	7% Tre	10% Tre
0-7	Daily gain (g/broiler/cage)	19.57	21.78 ± 0.39	20.54 ± 0.76	21.79 ± 0.39	19.69 ± 0.90	19.83 ± 0.66
	Daily feed intake (g/broiler/cage)	23.29	21.61 ± 0.26	19.41 ± 1.22	21.39 ± 0.34	20.29 ± 1.21	20.51 ± 0.64
	Feed conversion ratio (g feed/g wt.)	1.19	0.99 ± 0.01	0.94 ± 0.04	0.98 ± 0.01	1.04 ± 0.01	1.03 ± 0.01
0-14	Daily gain (g/broiler/cage)	29.14	35.59 ± 0.95	34.49 ± 1.03	36.01 ± 0.90	34.43 ± 1.17	35.05 ± 1.06
	Daily feed intake (g/broiler/cage)	37.71	39.50 ± 2.17	37.38 ± 1.19	39.94 ± 0.61	38.42 ± 1.28	39.27 ± 1.02
	Feed conversion ratio (g feed/g wt.)	1.29	1.11 ± 0.03	1.08 ± 0.01	1.11 ± 0.01	1.12 ± 0.01	1.12 ± 0.00
0-21	Daily gain (g/broiler/cage)	39.33	48.60 ± 1.43	47.17 ± 1.44	48.69 ± 1.24	48.00 ± 1.52	48.19 ± 1.28
	Daily feed intake (g/broiler/cage)	55.19	59.51 ± 1.59	56.78 ± 1.52	59.76 ± 1.54	58.42 ± 1.94	58.79 ± 1.54
	Feed conversion ratio (g feed/g wt.)	1.40	1.22 ± 0.01	1.20 ± 0.01	1.23 ± 0.02	1.22 ± 0.01	1.23 ± 0.01
0-28	Daily gain (g/broiler/cage)	48.71	60.40 ± 1.93	60.53 ± 1.77	61.75 ± 1.79	60.12 ± 1.90	61.26 ± 1.66
	Daily feed intake (g/broiler/cage)	74.29	78.48 ± 2.62	78.00 ± 1.71	80.91 ± 3.67	78.67 ± 1.41	79.50 ± 2.23
	Feed conversion ratio (g feed/g wt.)	1.53	1.30 ± 0.02	1.29 ± 0.02	1.31 ± 0.04	1.31 ± 0.03	1.31 ± 0.02
0-35	Daily gain (g/broiler/cage)	56.3	69.07 ± 2.03	70.22 ± 1.97	73.24 ± 2.03	69.97 ± 2.01	70.06 ± 1.68
	Daily feed intake (g/broiler/cage)	93.31	97.42 ± 2.73	97.49 ± 1.55	104.27 ± 2.20	97.51 ± 1.41	97.42± 2.73
	Feed conversion ratio (g feed/g wt.)	1.66	1.41 ± 0.05	1.39 ± 0.02	1.42 ± 0.03	1.40 ± 0.02	1.40 ± 0.06
0-35	Mortality (number of broilers)	-	0	0	0	1	1

The data are given as mean±SEM (n = 3). Mean values with different letters in each testing parameter with the same feeding period are significantly different ($p < 0.05$). No significant differences ($p > 0.05$) were observed in daily weight gain, feed intake or feed conversion ratio among the groups with 3%, 5%, 7% and 10% trehalose supplementation when compared to the control group throughout the test period. [1] Data are offered by the broiler feeding manual of Aviagen, a commercial broiler breeding company.

Supplementary Table S9. Effects of trehalose (Tre) on body weight (g) of broilers orally challenged with . *jejuni* (C.J.).

		Treatment				
**Age (days)**	**Aviagen[1]**	**Control**	**0% Tre+C.J.**	**1% Tre+C.J.**	**3% Tre+C.J.**	**5% Tre+C.J.**
0	42.00	45.32± 0.66	44.64 ± 0.77	45.31 ± 0.78	44.96 ± 0.62	45.15 ± 0.71
7	179.60	193.92 ± 3.46	191.24 ± 3.77	187.89 ± 3.13	191.26 ± 3.48	184.90 ± 3.54
14	450.00	517.24 ± 9.51	515.07 ± 12.90	514.86 ± 10.00	507.37 ± 11.97	502.15 ± 7.81
21	868.00	1001.89 ± 20.87	1001.12 ± 22.05	1020.21 ± 21.47	1002.25 ± 26.82	966.53 ± 15.24
28	1406.00	1492.94 ± 34.75	1527.11 ± 35.82	1545.68 ± 25.66	1579.23 ± 39.76	1532.00 ± 27.27
35	2013.00	2108.29 ± 48.77	2063.19 ± 49.67	2146.26 ± 51.31	2186.96 ± 47.30	2066.52 ± 33.47

The data are given as mean±SEM (n = 14~15). Mean values with different letters in each feeding week were significantly different ($p < 0.05$). Body weight of broilers showed no significant differences ($p > 0.05$) between *C. jejuni* and trehalose supplementation in both pre-inoculation and post-inoculation periods. [1] Data are offered by the broiler feeding manual of Aviagen, a commercial broiler breeding company.

Supplementary Table S10. Effects of trehalose (Tre) on daily weight gain and feed intake, feed conversion ratio and mortality of broilers orally challenged with *C. jejuni* (C.J.) according to pre-inoculation and post-inoculation periods.

Feeding period (day)	Parameter	Aviagen[1]	Control	Treatment 0%Tre+C.J.	1%Tre+C.J.	3%Tre+C.J.	5%Tre+C.J.
				**Pre-inoculation period**			
0-28	Daily gain (g/broiler/cage)	48.71	49.91 ± 0.68	52.31 ± 0.82	54.31 ± 0.36	53.11 ± 2.84	51.41 ± 1.38
	Daily feed intake (g/broiler/cage)	74.29	70.71 ± 1.23	73.68 ± 1.94	76.35 ± 0.50	75.18 ± 4.40	72.54 ± 1.71
	Feed conversion ratio (g feed/g wt.)	1.53	1.42 ± 0.01	1.41 ± 0.02	1.41 ± 0.01	1.41 ± 0.01	1.41 ± 0.00
				**Post-inoculation period**			
28-35	Daily gain (g/broiler/cage)	86.71	83.39 ± 3.75	78.18 ± 4.54	85.54 ± 5.25	83.73 ± 3.14	81.27 ± 1.74
	Daily feed intake (g/broiler/cage)	169.43	143.17 ± 7.65	135.40 ± 8.89	148.41 ± 11.67	145.94 ± 13.16	141.92 ± 4.33
	Feed conversion ratio (g feed/g wt.)	1.95	1.72 ± 0.02	1.73 ± 0.07	1.73 ± 0.07	1.74 ± 0.12	1.75 ± 0.06
Overall (0-35)	Mortality (number of broilers)	-	1	0	0	0	0

The data are given as mean±SEM (n = 3). Mean values with different letters in each feeding week were significantly different ($p < 0.05$). The average daily weight gain, feed intake and feed conversion ratio showed no significant differences ($p > 0.05$) between *C. jejuni* and trehalose supplementation in both pre-inoculation and post-inoculation periods. [1] Data are offered by the broiler feeding manual of Aviagen, a commercial broiler breeding company.

Supplementary Table S11. Effects of trehalose (Tre) on total *Lactobacillus* counts in portions of intestine or feces of broilers orally challenged with *C. jejuni* (C.J.)

**Intestinal Segment /Faces**	**Treatment**				
	**Control**	**0%Tre+C.J.**	**1%Tre+C.J.**	**3%Tre+C.J.**	**5%Tre+C.J.**
	**Total *Lactobacillus* counts (log cfu/g intestinal content or feces)**				
Duodenum	10.46 ± 0.34ab	9.72 ± 0.39b	10.64 ± 0.33ab	11.63 ± 0.39a	10.66 ± 0.34ab
Jejunum	11.71 ± 0.19a	11.49 ± 0.26a	11.80 ± 0.22a	11.40± 0.41a	11.65 ± 0.23a
Ileum	11.35 ± 0.24c	11.31 ± 0.20c	11.80 ± 0.22bc	12.15 ± 0.16a	12.09 ± 0.25ab
Caecum	10.75 ± 0.29b	10.78 ± 0.37b	11.51 ± 0.32ab	11.95 ± 0.23a	11.18 ± 0.26ab
Feces	12.74 ± 0.19a	12.90 ± 0.10a	12.63 ± 0.30a	12.34 ± 0.26a	13.06 ± 0.19a

The data are given as mean±SEM (n = 14~15, except feces n = 3 with at least triplicate per cage). Mean values within each tested portion of intestine or feces with different letters are significantly different ($p < 0.05$)

Supplementary Table S12. Effects of trehalose (Tre) on body weight (g) of broilers orally challenged with *C. perfringens* (C.P.).

		Treatment					
**Age(day)**	**Aviagen[1]**	**Control**	**0% Tre+C.P.**	**1% Tre+C.P.**	**3% Tre+C.P.**	**5% Tre+C.P.**
0	42.00	45.32± 0.66	42.71 ± 0.64	43.39 ± 0.64	43.33 ± 0.57	43.28 ± 0.61
7	179.60	193.92 ± 3.46	196.13 ± 4.09	200.36 ± 4.14	194.76 ± 3.10	198.91 ± 4.18
14	450.00	517.24 ± 9.51	463.56 ± 8.30	462.15 ± 9.30	462.13 ± 8.68	466.54 ± 10.45
21	868.00	1001.89 ± 20.87	912.35 ± 16.56	904.22 ± 21.21	900.91 ± 15.89	877.91 ± 22.22
28	1406.00	1492.94 ± 34.75	1418.66 ± 30.99	1434.39 ± 27.36	1454.35 ± 31.50	1418.43 ± 42.04
32	1748.00	1790.38 ± 19.47	1794.44 ± 54.38	1795.36 ± 40.75	1819.35 ± 54.99	1845.40 ± 56.64
39	2370.00	2492.59 ± 61.55	2424.71 ± 60.18	2523.09 ± 53.09	2464.07 ± 60.79	2497.58 ± 108.58

The data are given as mean±SEM (n = 13~15). Mean values with different letters in each feeding week were significantly different ($p<0.05$). Body weight of broilers showed no significant differences ($p > 0.05$) between *C. perfringens* and trehalose supplementation in both pre-inoculation and post-inoculation periods. [1] Data are offered by the broiler feeding manual of Aviagen, a commercial broiler breeding company.

Supplementary Table S13. Effects of trehalose (Tre) on daily weight gain and feed intake, feed conversion ratio and mortality of broilers orally challenged with *C. perfringens* (C.P.) according to pre-inoculation and post-inoculation periods.

Feeding period (day)	Parameter	Aviagen[1]	Control	Treatment 0%Tre+C.P.	1%Tre+C.P.	3%Tre+C.P.	5%Tre+C.P.
				**Pre-inoculation period**			
0-32	Daily gain (g/broiler/cage)	53.31	54.69 ± 0.52	54.81 ± 0.56	54.72 ± 0.37	55.66 ± 1.55	56.51 ± 0.80
	Daily feed intake (g/broiler/cage)	85.28	79.75 ± 1.07	81.02 ± 1.18	79.68 ± 0.85	81.25 ± 2.39	81.79 ± 1.12
	Feed conversion ratio (g feed/g wt.)	1.60	1.46 ± 0.01	1.48 ± 0.01	1.46 ± 0.01	1.46 ± 0.01	1.45 ± 0.00
				**Post-inoculation period**			
33-39	Daily gain (g/broiler/cage)	88.90	88.58 ± 5.70	93.42 ± 0.46	103.97 ± 1.64	96.14 ± 6.22	94.30 ± 4.50
	Daily feed intake (g/broiler/cage)	187.14	161.89 ± 8.13	168.38 ± 0.64	186.57 ± 6.36	171.20 ± 8.94	172.26 ± 4.21
	Feed conversion ratio (g feed/g wt.)	2.11	1.83 ± 0.06	1.80 ± 0.01	1.79 ± 0.04	1.79 ± 0.04	1.83 ± 0.05
Overall (0-39)	Mortality (number of broilers)	-	0	1	1	1	2

The data are given as mean±SEM (n = 3). Mean values with different letters in each feeding week were significantly different ($p < 0.05$). The average daily weight gain, feed intake and feed conversion ratio showed no significant differences ($p > 0.05$) between *C. perfringens* and trehalose supplementation in both pre-inoculation and post-inoculation periods. [1] Data are offered by the broiler feeding manual of Aviagen, a commercial broiler breeding company

Supplementary Table S14. Effects of trehalose (Tre) on total *Lactobacillus* counts in portions of intestine or feces of broilers orally challenged with *C. perfringens* (C.P.).

**Intestinal Segment /Faces**	**Treatment**				
	**Control**	**0%Tre+C.P.**	**1%Tre+C.P.**	**3%Tre+C.P.**	**5%Tre+C.P.**
	**Total *Lactobacillus* counts (log cfu/g intestinal content or feces)**				
Duodenum	10.85 ± 0.38a	9.49 ± 0.29b	10.62 ± 0.18a	10.47 ± 0.27a	10.88 ± 0.41a
Jejunum	11.32 ± 0.16a	11.20 ± 0.16a	11.02 ± 0.13a	11.17± 0.20a	11.03 ± 0.18a
Ileum	11.70 ± 0.16a	10.96± 0.10b	11.00 ± 0.14b	11.62 ± 0.14a	11.68 ± 0.13a
Caecum	10.49 ± 0.19a	10.48 ± 0.28a	10.23 ± 0.29a	9.87 ± 0.26a	10.14 ± 0.31a
Feces	12.22 ± 0.18a	12.76 ± 0.06a	12.46 ± 0.15a	12.59 ± 0.16a	12.74 ± 0.16a

The data are given as mean±SEM (n = 13~15, except feces n = 3 with at least triplicate per cage). Mean values within each tested portion of intestine or feces with different letters are significantly different ($p < 0.05$).
